# Red Fluorescent *Chlamydia trachomatis* Applied to Live Cell Imaging and Screening for Antibacterial Agents

**DOI:** 10.3389/fmicb.2018.03151

**Published:** 2018-12-18

**Authors:** Sergio A. Mojica, Anna U. Eriksson, Rohan A. Davis, Wael Bahnan, Mikael Elofsson, Åsa Gylfe

**Affiliations:** ^1^Department of Clinical Microbiology, Umeå University, Umeå, Sweden; ^2^Chemical Biology Consortium Sweden, Laboratories of Chemical Biology, Umeå University, Umeå, Sweden; ^3^Griffith Institute for Drug Discovery, Griffith University, Brisbane, QLD, Australia; ^4^Department of Molecular Biology, Umeå University, Umeå, Sweden; ^5^Department of Chemistry, Umeå University, Umeå, Sweden; ^6^Molecular Infection Medicine Sweden, Umeå University, Umeå, Sweden; ^7^Umeå Centre for Microbial Research, Umeå University, Umeå, Sweden

**Keywords:** *Chlamydia trachomatis*, high content screening, Australian natural products, antibacterial, anti-chlamydial, mCherry, fluorescence-based screening

## Abstract

In this study, we describe the application of a transformed *Chlamydia trachomatis* strain constitutively expressing the red fluorescent protein mCherry, to allow real-time monitoring of the infection cycle and screening for agents that block replication of *C. trachomatis*. The red fluorescent *C. trachomatis* strain was detected autonomously without antibody staining and was equally susceptible to doxycycline as the wild type strain. A high-throughput screening assay was developed using the transformed strain and automated fluorescence microscopy. The assay was used in a pilot screen of a 349 compound library containing natural products from Australian flora and fauna. Compounds with anti-chlamydial activity were tested for dose response and toxicity to host cells and two non-toxic compounds had 50% effective concentration (EC_50_) values in the low micromolar range. Natural products are valuable sources for drug discovery and the identified *Chlamydia* growth inhibition may be starting points for future drug development. Live cell imaging was used to visualize growth of the red fluorescent *C. trachomatis* strain over time. The screening assay reduced workload and reagents compared to an assay requiring immunostaining and could further be used to monitor the development of *Chlamydia* inclusions and anti-chlamydial effect in real time.

## Introduction

*Chlamydia trachomatis* is a major human pathogen causing sexually transmitted infections, infertility, trachoma, and blindness globally (Batteiger, 2012). According to the World Health Organization (WHO), 85 million people received antibiotics for trachoma, a blinding eye infection occurring in 42 countries (WHO, 2018), and there are more than 100 million annual cases of sexually transmitted *C. trachomatis* worldwide (Newman et al., 2015). Treatment options for infections with these obligate intracellular bacteria are limited and a single dose of azithromycin is the most common treatment worldwide. This regimen has selected for macrolide resistance in other human pathogenic bacteria (Ison, 2012; Bojang et al., 2017) and a more selective treatment would therefore be preferable. *C. trachomatis* readily develop resistance to antibiotics *in vitro* (Hammerschlag and Kohlhoff, 2012) and novel anti-chlamydial agents may become necessary for treatment of these infections in the future.

*Chlamydia spp*. replicate inside a specialized membrane-bound vacuole, the *Chlamydia* inclusion, and undergo a unique developmental cycle transitioning between two distinct bacterial forms; the elementary body (EB) is spore-like, infectious but metabolically limited, whereas the reticulate body (RB) is non-infectious but metabolically active. *Chlamydia* are protected by multiple lipid membrane barriers that reduce access for small molecules. Therefore, a whole cell screening assay for identification of novel anti-chlamydial agents is key to assure that active compounds reach the intracellular target and act in an intracellular infection. Several classes of antibacterial compounds that inhibit *Chlamydia* have been identified by screening using cell-based infection assays (Muschiol et al., 2006; Bailey et al., 2007; Dahlgren et al., 2010; Enquist et al., 2012; Hanski et al., 2014; Marwaha et al., 2014; Hakala et al., 2015; Sunduru et al., 2015; Good et al., 2016, 2017). The strict intracellular lifestyle of *Chlamydia* makes genetic manipulations challenging and genetically modified strains have, to the best of our knowledge not been used for small molecule screening in *Chlamydia*. Recent advances in genomics and *Chlamydia* genetics via novel transformation and genetic manipulation methods make this bacterium primed for further investigation (Wang et al., 2011; Bauler and Hackstadt, 2014). Screening for new anti-chlamydial drugs or tool compounds to study the infection is a tedious work involving multiple steps using toxic fixatives and multistep immunostaining. Since, the first fluorescent protein, green fluorescent protein (GFP) was cloned, numerous cells expressing fluorophore-coupled proteins have been described (Rodriguez et al., 2017). This has revolutionized live cell and animal imaging and simplified the study of intracellular processes. The development of truncated versions of GFP emitting at longer wavelengths, as well as the discovery of additional non-toxic fluorescent-proteins like the red fluorescent mCherry further improved the possibilities to study live cells (Shu et al., 2006).

Natural products have been used in both traditional medicine and modern drug discovery and have resulted in the development of numerous important clinical drugs (Li and Vederas, 2009). Due to the high levels of biodiversity found in both the terrestrial and marine ecosystems of Australia the use of this country's flora and fauna for both drug discovery and chemical biology has a strategic advantage, since this unique and chemically diverse resource has only been superficially explored for new pharmaceutical agents and chemical probes.

In this study, we describe the application of a transformed *C. trachomatis* strain constitutively expressing mCherry to an anti-chlamydial screen and demonstrate proof of concept with a 349 Australian natural compound library.

## Materials and Methods

### Organisms and Cell Lines

HeLa 229 cells (CCL-2.1; ATCC, Manassas, VA, USA) and Vero cells (CCL-81; ATCC, Manassas, VA, USA) were propagated in T75 flasks at 37°C with 5% CO_2_ in Roswell Park Memorial Institute 1640 (RPMI 640) medium or Dulbecco's Modified Eagle's medium (DMEM), respectively, supplemented with 10% Fetal Bovine Serum (FBS), 2 mM L-glutamine and 5 μg/ml gentamicin. *C. trachomatis* serovar L2 454/Bu (ATCC, Manassas, VA, USA) was propagated in HeLa cells and elementary bodies were purified as previously described (Caldwell et al., 1981) and stored in SPG buffer (0.25 M sucrose, 10 mM sodium phosphate, 5 mM L-glutamic acid) at −80°C.

### *Chlamydia* Transformation

The plasmid pBOMB4R-MCI designed for expression of the red fluorescent protein mCherry in *Chlamydia* was a generous gift from Ted Hackstadt (Laboratory of Intracellular Parasites, Hamilton, MT, USA) (Bauler and Hackstadt, 2014). *C. trachomatis* L2 454/Bu was transformed as previously described by Ian Clarke with modifications (Wang et al., 2011; Bauler and Hackstadt, 2014). 8 × 10^6^ inclusion forming units (IFUs) of *C. trachomatis* EBs were incubated with 5 μg of plasmid DNA in 200 μL of CaCl_2_ buffer (10 mM Tris pH 7.4, 50 mM CaCl_2_) at room temperature for 30 min. 1.5 × 10^6^ of trypsonized Vero cells were subsequently added to the same tube in 200 μL of CaCl buffer and incubated for 20 additional min with occasional mixing. The entire mixture was added to one well of a 6-well plate along with 2 ml of DMEM. The cells were allowed to settle for 1 h, and then the plate was spun at 750 g for 30 min to help initiate infection. Penicillin G (1 unit/ml) was added directly to the culture medium 18 h post infection (hpi). After 48 h, the cells were harvested by the removal of culture medium followed by the addition of 500 ml of dH_2_O for 10–15 min to lyse the cells. The lysate was carefully collected and spun at 1,000 rpm for 5 min, then 500 μL of the supernatant was added to a fresh layer of Vero cells and spun at 750 g for 30 min to help initiate a new round of infection. The media was then replaced with fresh DMEM containing 1 unit/ml penicillin G. Inclusions expressing mCherry appeared in passages 2–5 with 1 unit/ml penicillin G and were plaque purified into clones. Briefly, harvested bacteria were diluted in Hank's balanced salt solution (HBSS) and used to infect Vero cells in 6-well plates. After the infection, the inoculum was removed and the cells were overlaid with 0.54% agarose in DMEM supplemented with 10% FBS, 1 X non-essential amino acids, 10 μg/ml gentamicin, and 0.2 μg/ml cyclohexamide and incubated at 37°C with 5% CO_2_ for 10–14 days until plaques had formed. Plaques were harvested using a sterile 1 ml pipet tip, transferred to HBSS and used to infect fresh monolayers of HeLa cells in 24-well plates. One of the mCherry clones with good growth was amplified in HeLa cells, purified and stored as described above.

### Detection of mCherry Expressing *Chlamydia*

HeLa 299 cells, cultured to 70% confluence were harvested and seeded into 96-well plates (clear, flat, Nunclon Delta coated, Nunc, Thermo Fischer Scientific, Waltham, MA, USA) at a density of 10,000 per well in culture media (DMEM or RPMI 1640 supplemented with 10% FBS, 2 mM L-glutamine and 50 μg/ml gentamicin). After overnight incubation in 37°C with 5% CO_2_, adherent cells were infected with 30 μl mCherry expressing *Chlamydia* per well in HBSS with a multiplicity of infection (MOI) of 0.6. *Chlamydia* were incubated with the cells for 1 h at 37°C with 5% CO_2_ after which HBSS was removed and replaced with 100 μl culture media. DNA of host cell nuclei and *Chlamydia* inclusions were visualized by addition of 10 μl 10 μg/ml Hoechst 20 min prior to imaging. Live images were acquired 48 hpi in an automated HCS microscope (ArrayScan VTI, Thermo Fischer Scientific) using the 10x objective, excitation 549/15 nm for mCherry and excitation 386/23 nm for Hoechst. The number of large *Chlamydia* inclusions was enumerated using the built in software. An inclusion size cutoff was determined based on the 48 hpi *Chlamydia* inclusions to include normally developed *Chlamydia* inclusions and exclude fluorescent artifacts from the analyses. The cutoff also excluded small underdeveloped inclusions. To determine if detection of mCherry correlated with detection of immunostaining, cells were fixed and immunostained (Marwaha et al., 2014) after live cell acquisition. In brief, cells were fixed in 150 μl methanol per well for 10 min, blocked in phosphate buffered saline (PBS) supplemented with 1% bovine serum albumin (BSA) and 0.25% Tween-20, and stained with an in-house generated polyclonal rabbit anti-chlamydial serum (Marwaha et al., 2014) diluted 1:1,000 in blocking buffer. Finally, inclusions were visualized with a secondary donkey anti-rabbit fluorescein isothiocyanate (FITC) labeled antibody (Jackson ImmunoResearch, West Grove, PA, USA) and cell nuclei were visualized by 1 μg/ml Hoechst staining. Images were obtained using ArrayScan with the 10x objective, excitation 485/20 nm and excitation 386/23 nm for Hoechst. Large *Chlamydia* inclusions were enumerated as described for mCherry. To evaluate whether the mCherry expressing strain was as susceptible to doxycycline as the wild type strain (wt), we determined 50% effective concentrations (EC_50_) values for doxycycline in both strains. Serial 1:2 dilutions of doxycycline in 100 μl culture medium (400 nM to 6 nM doxycycline) were added to HeLa cells infected with either *Chlamydia* strain 1 hpi. After 48 h, live images were acquired from the mCherry while the wild type (wt) strain was immunostained as described above. Large inclusions were counted by Arrayscan and EC_50_ values determined using GraphPad Prism v.5 (GraphPad software, La Jolla, CA, USA) with non-linear regression.

The timepoint when mCherry expressing *Chlamydia* inclusions were detectable by Arrayscan was monitored using live infection time-lapse microscopy with micrographs every hour from 10 to 24 hpi using bright-field for autofocusing with the 10x objective. *Chlamydia* infection was cultured in phenol red free DMEM in live cell chamber at 37°C with 5% CO_2_, but otherwise performed as described above.

### Compound Library

The Davis open access natural product-based library (Zulfiqar et al., 2017) was used for the screening. Most of the 349 compounds have been isolated from Australian natural sources, and this library also contain semi-synthetic natural product analogs, as well as known commercial drugs or synthetic compounds inspired by natural products. The natural product isolation or semi-synthetic procedures for the majority of compounds in this unique library have been previously published (Davis, 2005; Barnes et al., 2010; Choomuenwai et al., 2012; Levrier et al., 2013). All compounds were >95% pure when submitted for storage. The 349 distinct compounds used in this study were dispensed as 5 mM and 2 mM dimethylsulfoxide (DMSO) solutions into 384-well polypropylene (PP) microtiter plates (Costar, New York, USA). Plates were heat sealed after dispensing and stored at −20°C.

### Screen

A general and more detailed protocol for the screening assay is provided in (Supplementary Data Sheet 1) and an overview of the screening method is given in Figure 3. Since inhalation of *C. trachomatis* aerosols can cause laboratory acquired infections (Sewell, 1995), all procedures involving handling of live infection in open plates were performed manually inside a class II safety cabinet using 8-channel electronic pipets (Rainin E4 XLS, Mettler Toledo, Barcelona, Spain) and an enclosed aspiration system (Intergra VacuSafe, Zizers, Switzerland). The supplementary protocol includes suggestions for optimization of the assay for automation and screening of larger compound libraries.

Prior to screen, the MOI was optimized by infecting HeLa cells with serial dilutions of the mCherry strain from MOI 7.5 to 0.056 in triplicate wells in 96-well plates as described in section Detection of mCherry Expressing *Chlamydia*. DMSO tolerance of the HeLa cells was determined by incubating 10,000 HeLa cells per well with 5, 4, 3, 2, 1, 0.5, and 0% DMSO in triplicate wells for 48 h in 37°C with 5% CO_2_. HeLa cell viability was measured by resazurin reduction using Presto-Blue (Thermofisher Scientific) according to the manufacturer's description. Fluorescence was measured in Synergy H1 plate reader (Biotek Instruments, Winooski, VT, USA) using 535/590 nm.

The primary screen of the 349 open access compound library was performed using the mCherry expressing *C. trachomatis* serovar L2 454/Bu in clear, flat 96-well plates (Nunc, Delta surface, Thermo Fischer Scientific). The primary screen was done in duplicates in separated plates; one plate was used for mCherry imaging and the other for FITC immunostaining. Cells were seeded at a density of 10,000 cells per well in 100 μl DMEM media 1 day prior to infection in culture media. *Chlamydia* infection was performed in 30 μl HBSS as described above with a MOI of 0.6 during 1 h at 37°C, 5% CO_2_. Compounds were diluted to 10 μM in DMEM with a final DMSO concentration of 0.5% and 100 μl per well was added after aspiration of HBSS. The plates were incubated for an additional 47 h at 37°C, 5% CO_2_ prior to imaging. One of the duplicate plates was used for mCherry imaging and one for FITC immunostaining. For mCherry imaging, HeLa cell nuclei were first stained by addition of 10 μl 10 μg/μl Hoechst per well and incubated at 37°C with 5% CO_2_ for 20 min. The infected cells were fixed in 4% paraformaldehyde (PFA) for 15 min and washed in PBS before image acquisition. Although fixation was not necessary for the analysis, it inactivated the infectious *Chlamydia* and enabled later analysis after storage at 4°C. For FITC immunostaining, cells were fixed in 150 μl methanol per well for 10 min, blocked in 1% BSA, 0.25% Tween-20 in PBS and incubated with 1:1,000 polyclonal rabbit anti-chlamydial serum in blocking buffer (Marwaha et al., 2014). A FITC-conjugated donkey anti-rabbit antibody (Jackson ImmunoResearch) and 1 μg/ml Hoechst staining was used for staining followed by washing with blocking buffer. Images of nuclei (Hoechst-ex386/23 nm) and *Chlamydia* inclusions (FITC: ex485/20 nm; mCherry: ex549/15 nm) were acquired in the ArrayScan with the 10x objective and counted with the built in software. *Chlamydia* inclusions in DMSO control wells were used to determine an inclusion size cutoff to exclude small underdeveloped inclusions as well as fluorescent artifacts from the analyses. Compounds with >95% inhibition of number of inclusion compared to the DMSO control and a cell nuclei count exceeding 3,000 were selected for further evaluation.

### XTT Reduction Assay

HeLa299 cells were inoculated into clear, flat 96-well plates (Nunc, Delta surface, Thermo Fischer Scientific) with a density of 7,000 cells per well and cultured overnight at 37°C with 5% CO_2_ in RPMI 640 supplemented with 10% FBS and 2 mM L-glutamine. The following day, the RPMI was replaced with DMEM without phenol red (Life Technologies, Carlsbad, CA, USA) containing 0.5% DMSO with or without test compounds at 25 and 50 μM concentrations incubated at 37°C with 5% CO_2_ for 48 h. At least three replicate wells for each compound concentration were tested. Wells without cells were used as blank controls. Following this incubation with compounds, cells were washed with DMEM to remove remaining colored compounds and fresh DMEM without phenol red was added before the XTT Cell Proliferation Assay kit was used according to the manufacturer's instructions (ATCC, Manassas, VA, USA). Briefly, activation reagent and XTT reagent were thawed at 37°C, then mixed in a 1:50 ratio with enough volume to add 50 μL to each well. The plates were placed in the 37°C incubator for 2.5 h. The absorbance of each well was then read at 475 nm and 660 nm with a Tecan Infinite m200 plate reader (Tecan, Männedorf, Switzerland). To analyze the data, the 660 nm reading was subtracted from the 475 nm reading to help eliminate non-specific readings from the assay results. Average blank control-well readings were subtracted from those containing cells. Final readings from wells containing compound were compared to DMSO control wells and expressed as percent of control.

### EC_50_ Determination

HeLa cells were inoculated into 96-well plates (Nunc, Corning, New York, USA) with a density of 15,000 cells per well and cultured overnight at 37°C with 5% CO_2_ in RPMI 1640, supplemented with 10% FBS and 2 mM L-glutamine, and infected the following day with wt or mCherry *C. trachomatis* in HBSS. At 1 hpi, the inoculum was replaced with RPMI media containing 0.5% DMSO and serial 1:2 dilutions of the tested compounds. The infection was allowed to proceed for 44–48 h before fixation by aspiration of the media and adding methanol for 5 min. *Chlamydia* inclusions were immunostained as described above. The numbers of large inclusions were counted by the ArrayScan VTI HCS automated scanner. Inhibition of *Chlamydia* growth was evaluated as the number of inclusions in compound treated infections (evaluated in 12 fields per well, 20x objective) compared to the numbers of inclusions in DMSO treated control infections (% of control). EC_50_ values are representative of at least three independent experiments. The data analysis was performed using non-linear regression (curve fit) in GraphPad Prism v.5.

### Live Real Time Imaging of *C. trachomatis* Infection With Compound 6

The anti-chlamydial effect over time of compound **6** (identified in the above screen) was visualized by a time-lapse experiment. HeLa cells were seeded at a concentration of 10,000 cells per well 1 day prior to infection. *Chlamydia* infection was performed as described above at MOI 0.6 with mCherry transformed *C. trachomatis*. Phenol red free DMEM culture media containing 6 or 3 μM **6** or 0.5% DMSO was added at 100 μl per well 1 hpi. Images were acquired with 1 h intervals with the 10x objective acquiring 4 fields per well during 48 h. The red channel was used for autofocusing and therefore imaging was initialized 24 hpi when the mCherry expressing inclusions were detectable, continuing to 72 hpi.

## Results

### The Red Fluorescent *C. trachomatis* Strain Can be Detected Autonomously Without Antibody Staining

**Figure 1:**
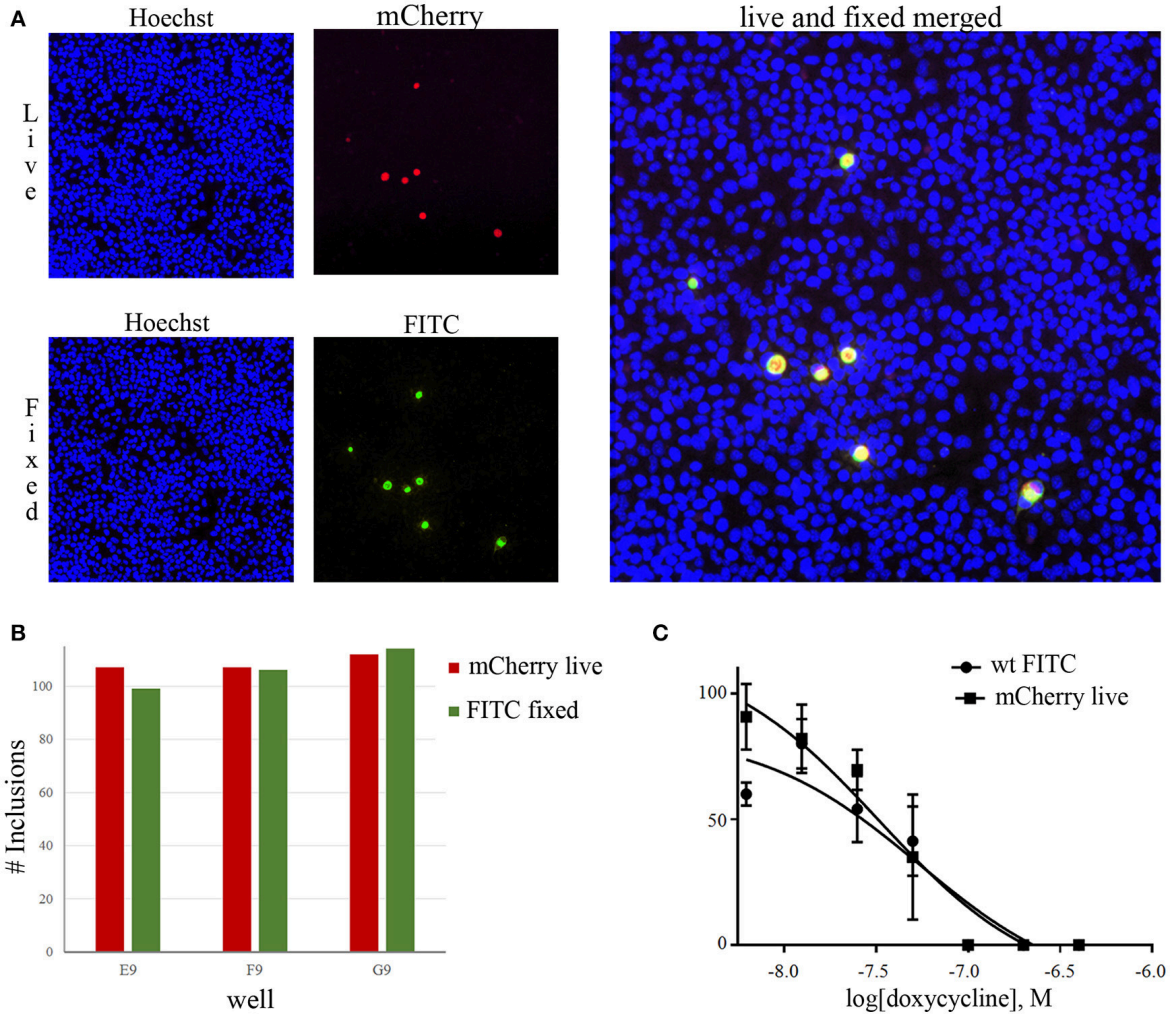
*C. trachomatis* expressing the red fluorescent protein mCherry can be detected without immunostaining. Images in **(A)** represents live infection imaging (upper) of Hoechst (blue) and mCherry (red). Lower panel images are acquired in the same field after immunostaining with FITC-labeled antibody. A merge of live and stained images is shown. Bars in **(B)** show the inclusion count obtained in in three individual wells from mCherry live imaging and FITC immunostained inclusions, respectively. **(C)** wt and mCherry expressing *C. trachomatis* are equally sensitive to doxycycline. Live imaging was used for mCherry expressing *C. trachomatis* inclusions while wt inclusions were visualized by FITC immunostaining.

To ensure automated detection of *C. trachomatis* inclusions by mCherry fluorescence alone is a feasible strategy, live images were acquired 48 hpi of HeLa cells infected with *C. trachomatis* transformed with pBOMB4R-MCI. After acquisition, the cells were fixed and immunostained with FITC-conjugated antibody and images of the same fields were acquired using the green channel. The mCherry and FITC signals overlapped exactly with negligible background (Figure 1A) when imaged using ArrayScan VTI HCS automated scanner. The automated scanner was also able to detect nearly exactly the same number of inclusions in the same wells from both mCherry and antibody staining (Figure 1B). The data from these quality control experiments demonstrate that mCherry signal alone adequately substitutes antibody staining for detection of *Chlamydia* inclusions. A time-lapse during the early development of *Chlamydia* showed that inclusions were detectable by mCherry after around 20 hpi (Supplementary Video 1). To verify that *C. trachomatis* transformed with pBOMB4-MCI is equally susceptible as the untransformed wt strain to benchmark antibiotics, we determined the EC_50_ of doxycycline against both the transformed and wt strain with similar results (Figure 1C). EC_50_ values with 95% confidence interval of wt and mCherry strains were 54.4 (18.7–158.0) and 32.8 (14.0–77.1), respectively, and thus, transformation with pBOMB4-MCI and expression of mCherry did not alter the susceptibility of *Chlamydia* to this antibiotic.

### Proof of Concept Screen of an Australian Natural Compound Library

**Figure 2:**
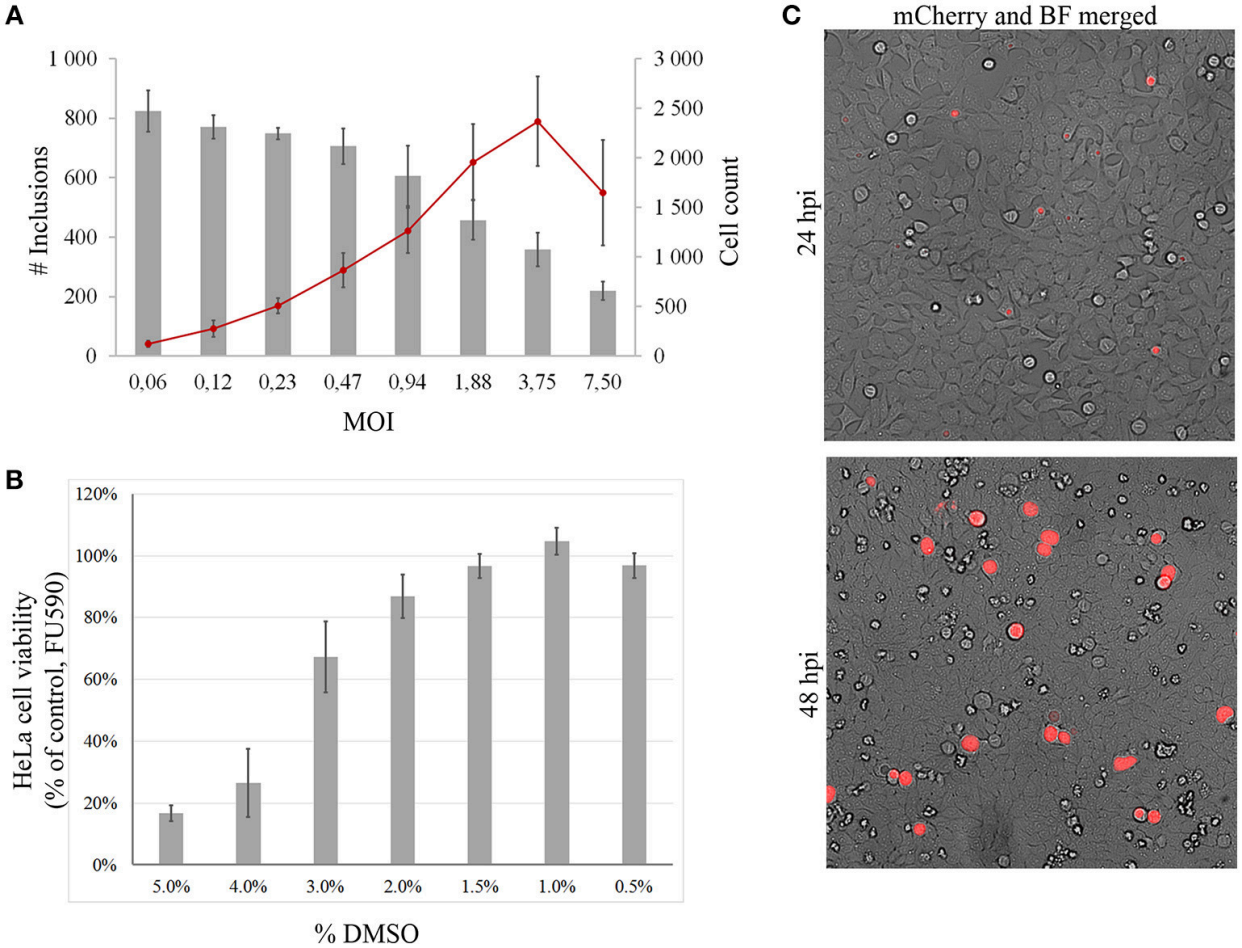
Assay optimization. **(A)** shows MOI titration with counted mCherry *Chlamydia* inclusions (red line, left y-axis) and counted HeLa cell nuclei (gray bars, right y-axis) at indicated MOI (x-axis). **(B)** DMSO tolerance of HeLa cells determined using a resazurin based cell viability assay quantified in fluorescence units at 590 nm (FU590). Bars represent % of control wells without DMSO. **(C)** The mCherry *Chlamydia* inclusions were big and easy to discriminate from artifacts at 48 hpi compared to small 24 hpi inclusions. Merged micrographs bright field (BF) and mCherry fluorescence.

As a proof of concept study with the transformed mCherry-expressing strain, we performed a screen of 349 compounds from the Davis open access Australian natural compound library (Zulfiqar et al., 2017). Assay optimization showed a linear increase of counted inclusions between MOI 0.1–1.0 with a probable overload at MOI 1 indicated as a loss of counted HeLa cell nuclei (Figure 2A). A MOI of 0.6 was chosen to obtain an adequate window for detecting *C. trachomatis* inhibitors within the linear increase of IFU with unaffected number of cells. Compounds were screened at a final concentration of 10 μM diluted from a 2 mM stock library with a final concentration of 0.5% DMSO which was well-tolerated by HeLa cells with no influence on their viability (Figure 2B). An incubation time of 48 h was chosen as the *Chlamydia* inclusions were large at the time point and easy to distinguish from possible artifacts (Figure 2C) by an inclusion size cut off.

**Figure 3:**
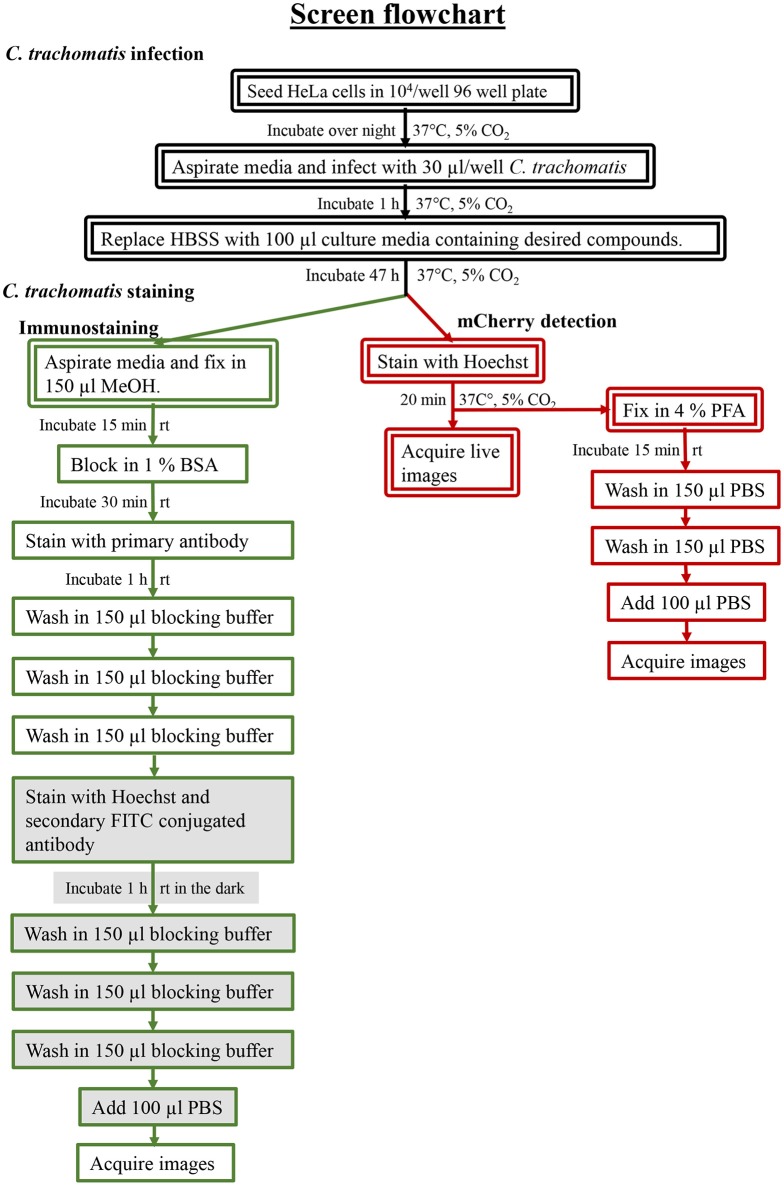
Screening workflow. The procedures are detailed in the Materials and Methods and a protocol is found in the Supplementary Material. Flowchart depict the different steps needed for the screening assay. Steps requiring safety cabinet are indicated with double lines. Steps used for mCherry imaging are highlighted in red and green lines indicate steps required for immunostaining with FITC. Filled textboxes indicate steps that needs to be performed in the dark.

**Figure 4:**
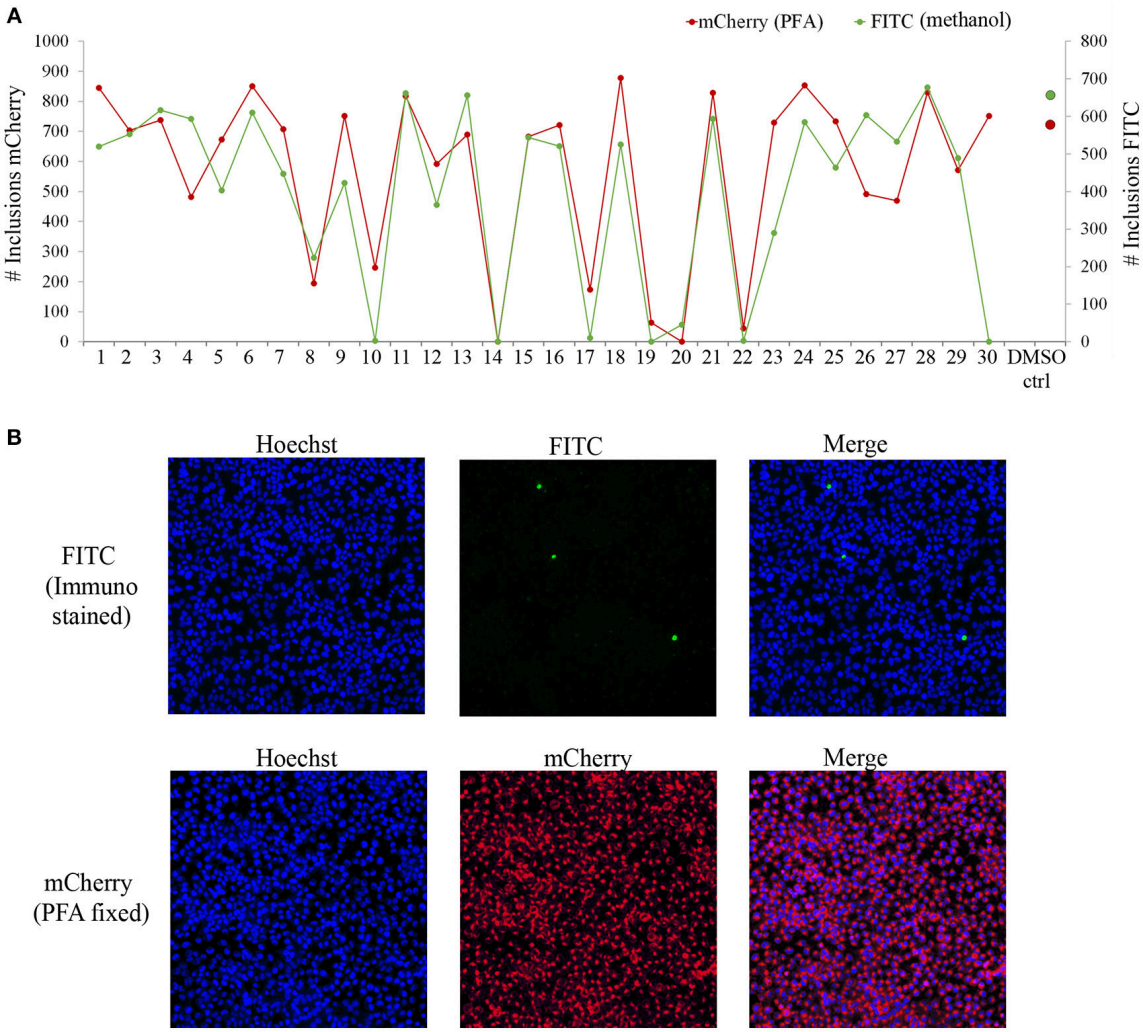
Screen data from 30 compounds. Plots in **(A)** show number of *Chlamydia* inclusions detected in the FITC-stained plate (green line) compared to those detected with mCherry (red line) for each compound in duplicate screen plates. Average inclusion counts obtained from DMSO control wells in FITC screen (green dot) and mCherry screen (red dot) are indicated. Images in **(B)** show one field in well 30 containing compound **3**, FITC-labeled (top) and the mCherry (bottom). Note how the compound stains the HeLa cells red obscuring the reduction in number of *Chlamydia* inclusions by the compound.

**Figure 5:**
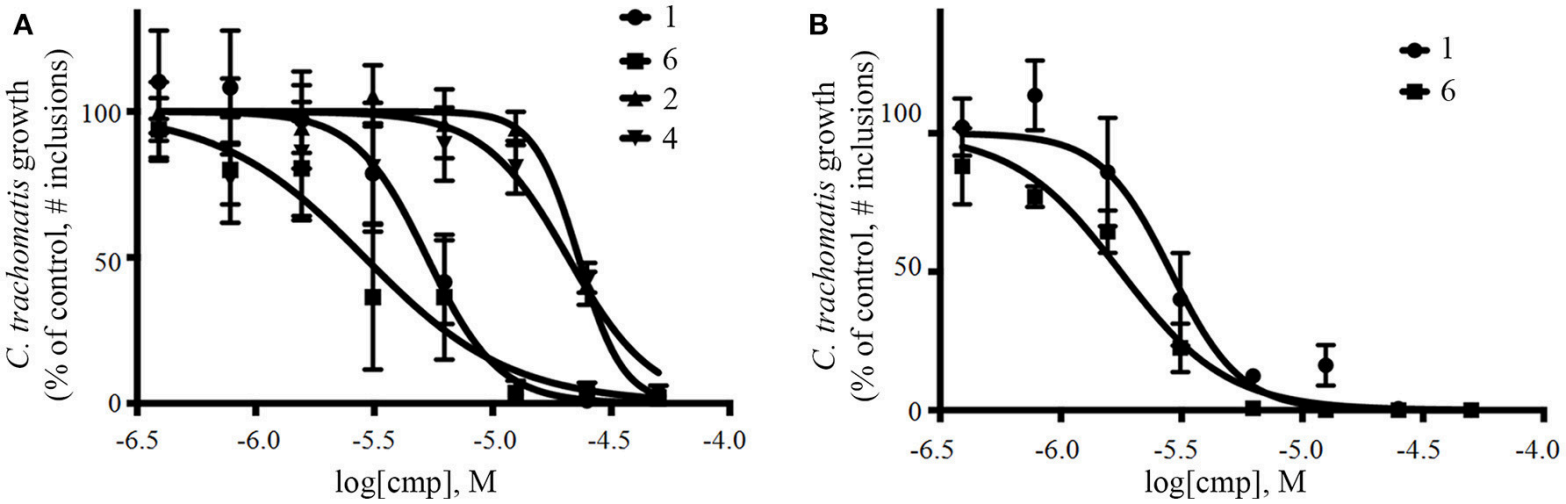
Dose-response curves of selected compounds against wt **(A)** and mCherry expressing **(B)** *C. trachomatis*. Both strains were visualized using FITC immunostaining. cmp, compound.

The screening was performed in duplicate plates and *Chlamydia* inclusions were detected either by mCherry-expression or by FITC-conjugated immunostaining. mCherry as readout significantly reduced the number of steps needed to perform the screen (Figure 3). The number of *Chlamydia* inclusions in the DMSO control wells correlated well between mCherry and FITC, with mean number of inclusions and standard deviations of 722 ± 61 and 657 ± 72, respectively. Z' was 0.75 for mCherry imaging and 0.62 for FITC imaging. The HeLa cell count as detected by Hoechst nuclear staining also showed a good correlation in the duplicate screens as expected (data not shown) and was used to exclude potentially toxic compounds from further analysis. A cutoff of >95% inhibition compared the infection control with DMSO was the first hit selection. As shown in Figure 4A there was an almost perfect correlation between the different detection methods. However, two compounds, **2** and **3** were only identified as hit compounds using FITC-immunostaining. Among these, **2** had 66% inhibition using mCherry while **3** showed no inhibition of *Chlamydia* growth using mCherry detection. A visual inspection of the acquired images revealed that the HeLa cells were bright red fluorescent in the well containing compound **3** thus obscuring the reduction in number of *Chlamydia* inclusions. In the follow-up dose response, these compounds were found to have a deep red color and a bright red auto-fluorescence staining the HeLa cells resulting in miss-identified *Chlamydia* inclusions in the automatic analysis as shown for **3** in Figure 4. However, two other red colored compounds (**1** and **4**), were correctly identified as hits using mCherry detection in the screen, but unfortunately the red fluorescence could not be used to enumerate *Chlamydia* at higher concentrations for any of the four red hit compounds. Finally, eight hit compounds with >95% inhibition of *Chlamydia* growth and a cell count exceeding 3,000 cells per well were selected for further analyses. Two additional compounds, **5** and **10** were also included in the further tests since the tetrahydroanthraquinone **5** is the parental natural product of the semi-synthetic red hit compounds **2**, **3**, and **4** (Choomuenwai et al., 2012; Barnes et al., 2016) while **10**, thiaplakortone A, is the parental natural product of the synthetic hit compounds **7**, **8**, and **9** (Schwartz et al., 2015). The selected compounds were evaluated for HeLa cell toxicity using the XTT reduction assay (Table 1). Of the eight hit compounds, only four compounds reduced cell viability < 69% at 25 μM compared to the DMSO control (Table 1). These four compounds were selected for evaluation with full EC_50_ determination with wt *C. trachomatis* (Figure 5A and Table 2). EC_50_ values were very similar when enumerating FITC stained mCherry and wt *Chlamydia* inclusions after treatment with the two most effective compounds, confirming that expression of mCherry did not alter response to antimicrobial compounds in *Chlamydia* (Figure 5 and Table 2).

**Table 1:** Selected compound after screening and HeLa cell viability in the XTT reduction assay.

**Compound ID**	**Structure**	**Screen inhibition at 10 μM (%) mCherry/FITC**	**HeLa cell viability at 25/50 μM (%)**
**1**	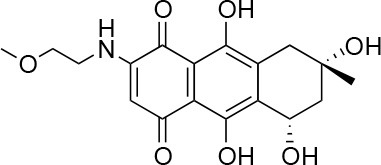	100/100	92.0 ± 8.0/58.5 ± 8.0
**2**	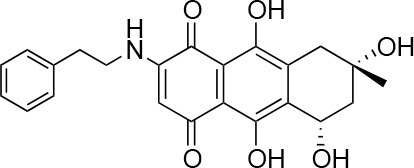	66/100	70.0 ± 13.0/31 ± 2.0
**3**	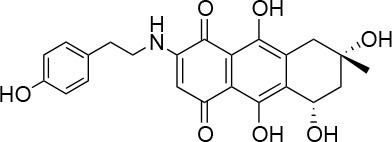	0/100	41.0 ± 2.5/24.0 ± 3.0
**4**	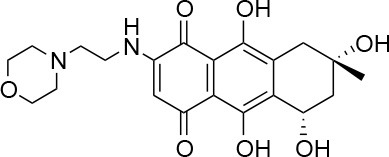	98/100	78.0 ± 6.0/74.0 ± 10.0
**5**	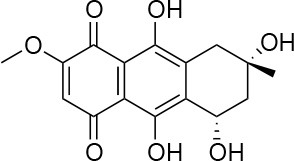	13/0	5 ± 0.5/5.5 ± 0.5
**6**	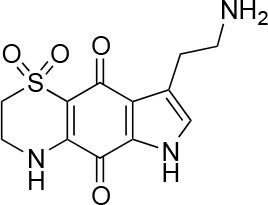	100/100	77± 0.2/55± 1
**7**	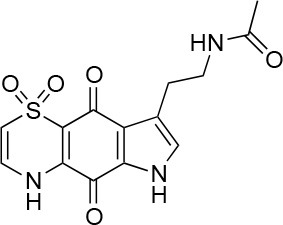	99/100	17.0± 2.0/6.0 ± 0
**8**	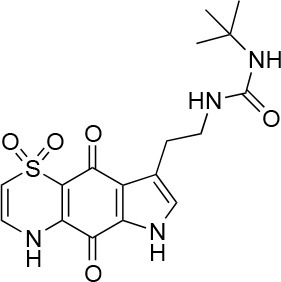	100/100	30.0 ± 7.0/11.0 ± 1.0
**9**	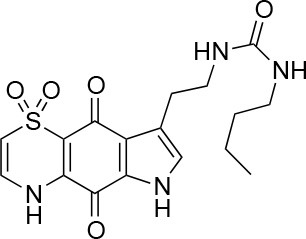	100/100	37 ± 6.5/11.5 ± 1
**10**	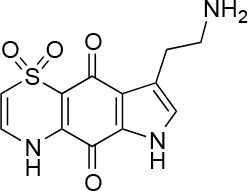	100/96	5.0 ± 0.4/5.0 ± 0.5

**Table 2:** EC_50_ values with 95% confidence values for selected compounds against wt and mCherry expressing *C. trachomatis*.

**Compound ID**	***Chlamydia* strain**	**EC_**50**_ (μM)**
1	wt	5.3 (4.5–6.2)
1	mCherry	2.8 (2.4–3.2)
2	wt	23.0 (21.3–25.0)
4	wt	20.8 (16.5–26.3)
6	wt	2.8 (2.2–3.8)
6	mCherry	1.7 (1.6–2.0)

### Live Cell Imaging of the *Chlamydia* Developmental Cycle

The red fluorescent strain was used to monitor the effect of the most effective compound **6** in real time over the infectious cycle. Using the live microscopy and incubator functions, images were obtained with the ArrayScan VTI HCS automated scan every hour between 24 and 72 hpi. The red fluorescent *Chlamydia* inclusions were not detectable at 6 μM **6** (data not shown) and grew at a slower pace with 3 μM **6** (Supplementary Video 2), compared to the DMSO control (Supplementary Video 3). The reduced inclusion size throughout the developmental cycle through to burst of inclusions suggest a continuous growth reduction in presence of **6**.

## Discussion

The technique to transform *C. trachomatis* with a modified plasmid has opened opportunities for different applications (Wang et al., 2011). We transformed *C. trachomatis* with a published plasmid (Bauler and Hackstadt, 2014) enabling expression of the red fluorescent protein mCherry. The transformed strain was applied to an assay detecting compounds that reduced the number of *Chlamydia* inclusions in cell culture by automated microscopy. This *Chlamydia* strain was equally susceptible to tested compounds compared to the wt strain demonstrating its usefulness in identifying novel inhibitory compounds. Hands on time was considerably reduced compared to visualization of *Chlamydia* inclusions by immunostaining that demands multiple steps of fixation, incubation with antibodies and wash steps while the red fluorescent strain could be visualized live without previous preparation. The screening protocol could be further automated given that the biosafety aspects are properly addressed for example by using liquid handling automation placed inside safety cabinets or closed systems. Screening using *Chlamydia* immunostaining protocols has been validated for different *Chlamydia* species (Osaka et al., 2012) and has identified different classes of anti-chlamydial compounds such as salicylacylidene acylhydrazides (Muschiol et al., 2006; Wolf et al., 2006; Ur-Rehman et al., 2012; Bao et al., 2014), 8-hydroxyquinoline based inhibitors (Enquist et al., 2012), *N*-acylated derivatives of sulfamethoxazole and sulfafurazole (Marwaha et al., 2014), salicylacylidene acylhydrazide sulfonamide hybrids (Sunduru et al., 2015), the *Chlamydia* protease inhibitor-peptide Boc-Val-Pro-ValP(OPh2) (JO146) (Gloeckl et al., 2013; Ong et al., 2015), the isoflavone biochanin A (Hanski et al., 2014), dibenzocyclooctadiene lignans (Hakala et al., 2015), 2-pyridones (Engström et al., 2014; Good et al., 2016, 2017) and 2,3-diarylbenzofuran and 2,3-dihydrobenzofuran based inhibitors (Saleeb et al., 2018). Screening protocols without immunostaining have also been developed previously, for example a protocol using a fluorescent lipid, 6{*N*-[(7-nitrobenzo-2-oxa-1,3-diazol-4-yl)-amino]caproylsphingosine}(WHO, 2018) (C6-NBD-ceramide) that is converted to fluorescent sphingomyelin and accumulates in *Chlamydia* inclusions (Sandoz et al., 2012). Another approach utilized resazurine reduction as readout based on that *Chlamydia* infected cells reduce resazurine less effectively compared to uninfected cells (Osaka and Hefty, 2013). While these assays have similar hands on time as the method we present, their respective read-out depends on host cellular functions while our method detected fluorescence directly produced by *Chlamydia*.

We screened a library of Australian natural compounds and identified the same hit compounds with mCherry as with immunostaining of *Chlamydia*, with two exceptions, **2** and **3**. These compounds were only selected as hit compounds using the green fluorescent immunostaining of *Chlamydia* inclusions and not using the red fluorescence. It appeared that these compounds had red color and red auto-fluorescence mimicking the signal from healthy *Chlamydia* inclusions, thereby obscuring the reduction of *Chlamydia* inclusions. This was also true for **1** and **4** in concentrations above the screening concentration. Natural compounds may be autofluorescent in the whole fluorophore spectrum (Garcïa-Plazaola et al., 2015) and thus screening of natural compounds with a fluorescent based assay may result in either failure to detect active compounds or false positive hits. A green or yellow fluorophore may be as prone to this error as a red one and screening at lower concentrations and using a lower cutoff could reduce this problem.

Isolation, screening and biological profiling of Australian natural compounds that possess anti-parasitic activity was recently reported (Zulfiqar et al., 2017). Here we identified two classes of Australian natural compounds that inhibited growth of *Chlamydia*, tetrahydroanthraquinone and thiaplakortone compounds. The tetrahydroanthraquinone (1*S*,3*S*)-austrocortirubin (**5**) is a deep red colored fungal metabolite that has been previously isolated from the taxonomically related Australian mushrooms, *Dermocybe splendida* (Elsworth et al., 1999) and *Cortinarius* sp. (Choomuenwai et al., 2012). Compound **5** has antibacterial activity against *Staphylococcus aureus* with minimum inhibitory concentration (MIC) of 100 μM (Beattie et al., 2016), and antimalarial activity against *Plasmodium falciparum* parasites (IC_50_ = 1.9 μM). This compound also has toxic effects on mammalian cells (Choomuenwai et al., 2012) (Wang et al., 2015) and in this study **5** reduced HeLa cell viability by 95% at 25 μM. Despite the apparent cytotoxicity, **5** did not inhibit *Chlamydia* growth. However, semi-synthetic *N*-substituted tetrahydroanthraquinone analogs of **5** (Barnes et al., 2016) were hit compounds in our screen. While one compound, **3**, was toxic, three analogs **1**, **2**, and **4** did not reduce cell viability below 70% of vehicle control at 25 μM and were considered non-toxic. Compound **1** had the most potent anti-chlamydial effect with low micromolar EC_50_.

The remaining hit compounds **7**, **6**, **8**, and **9** were all thiaplakortone compounds and **10** was also investigated due to its similar structure. Thiaplakortone A (**10**) and B (**6**) were first isolated from a Great Barrier Reef sponge *Plakortis lita*. These compounds have *in vitro* antimalarial effect at low nanomolar concentrations (Davis et al., 2013) and **10** also inhibits *Trypanosoma brucei* and *T. cruzi*, at low micromolar concentrations (Zulfiqar et al., 2017). Compound **10** is known to have moderate toxicity in mammalian cells while **6** was non-toxic (Davis et al., 2013). In this screen **6** was the most potent hit compound and was also non-toxic to HeLa cells. **10** and its synthetic analogs **7**, **8**, and **9** (Schwartz et al., 2015) were all toxic to HeLa cells and as *Chlamydia* are obligate intracellular pathogens the anti-*Chlamydia* effect was not possible to distinguish from the cytotoxicity. The molecular target and mode of action of thiaplakortone and analogs in parasites is not known and we can only speculate that the anti-chlamydial effect may be caused by an effect of a common eukaryotic cell target altering the function of the host cell to the disadvantage of *Chlamydia*. Alternatively, a specific bacterial target may also be possible given the low host cell toxicity of a few compounds.

The use of a natural compound library from relatively unexplored Australian plants and fungi is especially interesting from an antibiotic development perspective since pre-existing resistance mechanisms are less likely. The new *Chlamydia* inhibitors identified in the present screen have the potential to provide starting points for development of novel antimicrobial drugs. Treatment options for intracellular bacterial infections like *Chlamydia* are limited partly due to limited access to the restricted intracellular compartment. Resistance to antibiotics readily develops *in vitro* and clinical isolates with antibiotic resistance as well as cases of treatment failure have been described (Hammerschlag and Kohlhoff, 2012). Although antibiotic resistance is currently not widespread in the clinical setting, antibiotic resistant strains pose a potential threat as effect of treatment is not monitored and antibiotic susceptibility testing is not performed. Our novel image-based screening assay is a useful tool in identification of novel anti-chlamydial agents that could be used to treat and prevent infections that cause infertility and blindness globally.

The red fluorescent strain was also visualized by repeated micrographs throughout the developmental cycle using the live cell imaging function of the automated microscope. The effect of the most potent compound **6** as observed by live microscopy was shown to reduce *Chlamydia* inclusion size compared to untreated infections suggesting antibacterial growth inhibition properties of the compound. The live microscopy function demonstrated a valuable tool for determining in what part of the infectious cycle the compound has its effect and may replace multiple time course experiments.

Here we present an image-based screening method with red fluorescent *Chlamydia* that was autonomously detected by automated microscopy without the need for *Chlamydia* immunostaining. The method was applied in image-based screening to compounds that inhibit *Chlamydia* growth and identified Australian natural compounds with previously described antimicrobial activity. Further, the method was used for live-cell monitoring of *Chlamydia* infected cells over time and visualized the growth inhibitory effect of the most potent natural compound in *Chlamydia* cell culture infection.

## Data Availability Statement

The chemical structures of the 339 compounds that were not selected as hit compounds are not publicly available due to other ongoing research collaborations and possible intellectual propriety rights. However, all chemical structures needed to support the conclusions in the manuscript are shown in Table 1.

## Author Contributions

ÅG and ME contributed conception and design of the study. RAD provided the compound library. AUE performed the screening and follow up testing. SAM transformed *Chlamydia*, performed toxicity testing and determined EC_50_ values and the live microscopy assay. WB purified the transformed *Chlamydia* and compared the two different fluorescence detection methods. SAM, AUE, and ÅG wrote sections of the manuscript. All authors contributed to manuscript preparation and approved the submitted version.

### Conflict of Interest Statement

The authors declare that the research was conducted in the absence of any commercial or financial relationships that could be construed as a potential conflict of interest.

## Supplementary Material

Supplementary Data Sheet 1 Screening protocol.

Supplementary Video 1 Expression of mCherry in transformed *C. trachomatis*. Time-lapse from 10 to 24 hpi. mCherry becomes visible around 20 hpi.

Supplementary Video 2 Treatment with 3 μM hit compound **6** leads to a continuous growth reduction. mCherry transformed *Chlamydia* time-lapse from 24 to 72 hpi.

Supplementary Video 3 Treatment with 0.5% DMSO, control for video 2. mCherry transformed *Chlamydia* time-lapse from 24 to 72 hpi.

## Abbreviations

wt: wild type
hpi: hours post infection
EC_50_: 50% effective concentration.
