# Supplementary material for: Red Fluorescent Chlamydia trachomatis Applied to Live Cell Imaging and Screening for Antibacterial Agents
Source: Front Microbiol. 2018 Dec 18;9:3151. doi: 10.3389/fmicb.2018.03151 (PMC6305398; doi:10.3389/fmicb.2018.03151)
Supplement: Supplementary Data Sheet 1 — Screening protocol. [file Data_Sheet_1.docx]

**Screening protocol**

Inhalation of *C. trachomatis* aerosols can cause laboratory acquired infections (Sewell, 1995), and handling of infectious liquids should be performed in a class II safety cabinet (EN 12469 Certified). For this screen all work proceeding fixation of infections were performed manually using electronic multichannel pipettes and a 96 well aspiration system (Intergra VacuSafe, VWR, Sweden) inside a safety cabinet. The procedure may be automatized using smaller liquid handling systems that fits inside the safety cabinet or fully integrated systems. This protocol describes screening using 96-well plate format. The screen could likely be transferred to 384-well format using the mCherry strain with adjustments of volumes only.

Fixation is not necessary when detecting mCherry fluorescence but allows screening at later time points and inactivates the infectious *Chlamydia*. Paraformaldehyde fixation is preferable to methanol when detecting mCherry fluorescence since the latter reduces the fluorescence intensity of mCherry. When using methanol fixation prior to immunostaining, it is imperative to ensure that the cells don’t dry out and we recommend fast replacement of methanol with blocking buffer after fixation.

1. **Chlamydia infection.**
   1. **Cell seeding**
      1. Harvest HeLa 299 cells, cultured to 70% confluence, by trypsination. Count and resuspend to 10^5^ cells/ml in culture media (DMEM supplemented with pyruvate, 50 µM gentamycin and 10 % FBS).
      2. Seed 100µl/well into clear flat bottom 96-well assay plates (Nunc^TM^ Delta surface, Thermofisher) to reach a density of 10^4^ cells/well.
      3. Incubate overnight at 37 °C, 5% CO_2_.
   2. **Infection.**

The linear range of *Chlamydia* IFU counts needs to be determined by titration of the *Chlamydia* stock in each screening system prior to a screening. We used a ratio of *Chlamydia* IFUs to HeLa cells, i.e. multiplicity of infection (MOI), of 0.6.

- - 1. Quickly defrost an aliquot of frozen mCherry *C. trachomatis* stock and dilute to desired concentration (200 IFU/µl in this screen) in prewarmed Hanks balanced salt solution (HBSS, Life Technologies, Carlsbad, CA, USA).
    2. Aspirate culture media from assay plate.
    3. Add 30µl *C. trachomatis* suspension/well for a MOI of 0.6.
    4. Add 30µl HBSS/well to uninfected control wells.
    5. Incubate for 1h at 37 °C, 5% CO_2_.
  1. **Compound addition.**

Compound library should be diluted to 2-10 mM in DMSO, dispensed into 384-well polypropylene (PP) plates, and heat sealed for storage. We use a final concentration of 10 µM compound in the primary screens.

- - 1. For duplicate plates and a 2 mM library: Transfer 1.1 µl compounds from a 384-well compound plate into 96w microtiter plates (Costar 96V PP, ThermoFisher) using an 8-channel pipet. This will result in one 96well plate/384well plate quadrant. It is preferable to spin compound plates before removing the seal.
    2. Dilute the transferred compounds in 220 µl prewarmed culture media/well to reach a final compound concentration of 10 µM/well and 0.5 % DMSO.
    3. Remove excess *C. trachomatis* from assay plate by aspirating the HBSS.
    4. Transfer 100 µl/well growth medium with compound or DMSO from the premixed plates to the assay plates. Add 100 µl culture media supplemented with 0.5 % DMSO per well to control wells.
    5. Incubate for an additional 47 h at 37 °C, 5% CO_2_.

**2. Visualization and acquisition.**

The steps following fixation of *C. trachomatis* infection do no longer require use of Safety Cabinet.

2.1. **Using mCherry expression for imaging**

2.1.1. Dilute Hoechst to 10 µg/ml and add 10 µl/well to assay plate

2.1.3. Incubate for 20 min. at 37 °C, 5% CO_2_.

2.1.4 Optional fixation step for acquisition at a later timepoint or if plates need to be moved from biosafety level rooms: Remove culture medium and add 100 µl/well 4 % PFA in PBS, incubate 15 min. at RT, and wash with 3 x 150 µl PBS/well. Store at 4 °C until acquisition.

2.1.5. Acquire about 4 - 5 fields/well with the 10 x objective in an automatic microscope (in this protocol we used an ArrayScan VTI, ThermoFisher, Waltham, PA, USA). mCherry has an excitation maximum around 586nm, in this protocol we used x549/15 for mCherry positive *C. trachomatis* inclusions and x386/23 for nuclei.

- 1. **Visualizing using immunostaining.**

2.2.1 Remove culture medium and fix in 150 µl/well methanol/well for 10 min.

2.2.2. Block in 100 µl/well PBS supplemented with 1 % bovine serum albumin (BSA, Gibco) and 0.025 % Tween.

2.2.3. Dilute EB antisera 1:1000 in blocking buffer (in this manuscript we use an in-house generated, polyclonal rabbit anti-*Chlamydia* sera), and add 100 µl/well to assay plate.

- - 1. Wash with 3x 150µl PBS/well.
    2. Dilute secondary donkey anti-rabbit fluorescein isocyanate (FITC)-labeled antibody (Jackson ImmunoResearch, West Grove, PA, USA) 1:500 and Hoechst nuclear stain to 1 µg/ml in blocking buffer and add 100 µl/well to assay plate.
    3. Acquire images as above but use xʎ488 nm (x485/20 in the current screen) to detect FITC positive *C. trachomatis* inclusions instead of xʎ549-572 nm.
  1. **Hit selection and statistics**

2.3.1. Use a well with infected cells to set a cutoff for inclusion size representing successfully replicating *C. trachomatis* inclusions.

- - 1. Use nuclear count to approximate cell numbers/well. Cell count lower than 50 % of DMSO control indicate potential compound toxicity.
    2. To validate the screen, use average (Avg) and standard deviation (SD) of *Chlamydia* inclusion count obtained from the control wells to calculate Z’ as 1-(((3*SD_infected_) + (3*SD_uninfected_)) / (Avg_infected_) - (Avg_uninfected_)). Z’ values should preferably be above 0.5 for reliable hit identification.
    3. There are several statistical tools available to select or reject potential hit-compounds. A > 3SD increase/decrease compared to controls is recommended, but also fold increase or % of control are commonly used to find compounds with activity.

*Reference*

Sewell, D.L. (1995). Laboratory-associated infections and biosafety. *Clin Microbiol Rev* 8(3)**,** 389-405.
